# Pediatric eye emergency department activity during the first wave of Covid-19 pandemic

**DOI:** 10.1186/s13052-021-01167-5

**Published:** 2021-11-04

**Authors:** Elia Franzolin, Rosa Longo, Elena Gusson, Benjamim Ficial, Giorgio Marchini

**Affiliations:** 1 Ophthalmic Unit, Department of Neurosciences, Biomedicine and Movement Sciences, University of Verona, Verona, Italy; 2 Neonatal Intensive Care Unit, Department of Pediatrics, University Hospital of Verona, Verona, Italy

**Keywords:** COVID-19, Eye emergency department, Pediatric emergencies, Ophthalmic emergencies

## Abstract

**Background:**

We investigated the volume and the characteristics of pediatric eye emergency department (PEED) consultations performed at our tertiary eye center during the early months of the COVID-19 pandemic and we compared them to those carried out in the same time interval of the previous three years.

**Methods:**

Ophthalmic emergency examinations of patients aged ≤18 years old and done during the national COVID-19 lockdown (March 9th, 2020 – May 3rd, 2020) and in the corresponding date range of the previous three years (2017, 2018, and 2019) have been considered and reviewed. The following features were retrieved and analyzed: age, gender, duration and type of accused symptoms, traumatic etiology, and the discharge diagnosis.

**Results:**

136, 133, and 154 PEED visits have been performed respectively in 2017, 2018, and 2019, while 29 patients presented in 2020. Therefore, the volume of PEED activity decreased by 79.4% (*p* < 0.0001). Demographical and clinical characteristics were comparable to those of the pre-COVID period. Despite the absolute reduction in the number of traumas, urgent conditions increased significantly from 30.7 to 50.7% (*p* = 0.024).

**Conclusions:**

PEED activity decreased consistently after the onset of the pandemic and it was mainly attended by those children whose conditions required prompt assistance, reducing the number of patients diagnosed with milder pathologies. At the end of the emergency, better use of PEED could avoid overcrowding and minimize waste, allowing resource optimization for the management of urgent cases.

## Background

The COVID-19 pandemic has had significant consequences on the population worldwide, determining a social, economic, and medical crisis. Measures adopted to limit the contagion have influenced people’s habits, daily activities and lifestyle. Although subjects of any age are at risk of contracting the infection, patients who are immunocompromised or with comorbidities, and the elderly are more likely to develop a severe illness with complications [1]. Conversely, children and young adults seem to be rarely affected by the virus and by them the course of disease is often asymptomatic, although skin manifestations or anecdotal cases of Kawasaki syndrome were described [2]. As a consequence, school shut down and interruption of leisure and sport activities have been among the most common restrictions that national governments have introduced. To date, several studies have reported the discontinuity in health care assistance that has followed the outbreak. Clinicians have focused on the management of the emergency, making the handling of both chronic and acute pathologies complicated [3]. Even in the ophthalmic field, a significant drop of both scheduled and emergency consultations and surgeries has occurred, leading to worse visual outcomes for patients [4], if compared to the period before the pandemic. This study aims to investigate the volume and the characteristics of pediatric eye emergency department (PEED) consultations carried out in our center during the Italian national lockdown, highlighting the effects that the pandemic had driven. Our tertiary eye clinic is located in one of the ten cities most affected by the infection during the first wave, so it can be considered as representative of the national scenario.

## Methods

This is a retrospective observational study conducted in accordance with the tenets of the Declaration of Helsinki and it was previously approved by the Ethical Committee of our Institution. Informed consent for any medical treatment and data processing was given by parents or authorized legal guardians at the moment of presentation.

Ophthalmic emergency consultations of patients aged ≤18 years old and performed during the national lockdown (March 9th, 2020 – May 3rd, 2020) and in the corresponding period of the previous three years (2017, 2018, and 2019) have been considered and reviewed. The following characteristics were collected: age, gender, duration and type of accused symptoms, traumatic etiology, laterality and the discharge diagnosis. Each diagnosis has been then classified according to the interested ocular sub area and to urgency. Channa’s classification has been used to define urgency [5].

Since the samples of 2017, 2018, and 2019 did not differ significantly from each other for any of the collected variables, they have been considered as a whole for the statistical analysis, the so called pre-COVID-19 Group (pCG), and compared to the COVID-19 Group of patients that presented in 2020 (CG).

Descriptive results were reported as percentage, mean ± Standard Deviation (SD) and median with interquartile range (IQR) respectively for categorical, normally distributed and not-normally distributed variables. Differences between pCG and CG were assessed using Fisher Exact test for categorical variables, T-test for independent means to compare patients’ age, and Mann Whitney U-test for the number of daily consultations performed and symptoms’ duration. The analysis was done using STATA 16.0 (StataCorp, Texas, USA). Statistical significance was considered when *p* < 0.05.

## Results

**Fig. 1:**
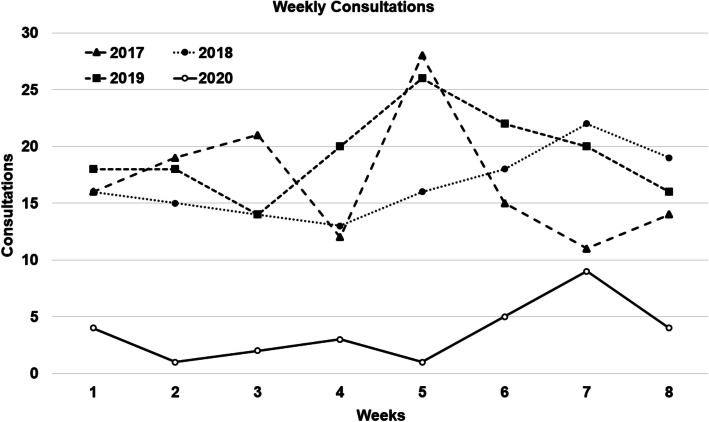
Average number of weekly PEED consultations performed in the considered date range of 2017, 2018, 2019, and 2020

136, 133, and 154 PEED consultations have been performed respectively in the considered period of 2017, 2018, and 2019, while 29 patients presented in 2020. So, during the national lockdown, the volume of PEED examinations decreased by 79.4%. The average number of weekly consultations in the different years is showed in Fig. 1.

Descriptive results and significances between pCG and CG are reported in Table 1. Overall, patients’ age and gender, duration of symptoms, etiology and pathological category did not significantly differ between the two periods. Redness, discomfort, swelling and pain confirmed as the most referred symptoms at presentation. A significance has been found in urgency classification, since urgent diagnosis increased from 30.7 to 50.7% in 2020 (*p* = 0.024). The commonest urgent conditions of the CG group were “corneal abrasion” (33%), “peri-ocular trauma” (20%), “traumatic hyphema” and “corneal foreign body” (both 13%), whereas the most frequent urgencies in the pCG group were “corneal abrasion” (33%), “peri-ocular trauma” (22%) and “corneal foreign body” (10%).

**Table 1:** Comparison of demographic and clinical characteristics of pCG and CG

	pCG (***n*** = 423)	CG (***n*** = 29)	***p***-value
**Patients per day**	2 (1–4)	0 (0–1)	**< .00001**
**Age, years**	8.38 ± 5.0	8.6 ± 5.6	.862
**Gender, F (%)**	38.8	48.3	.331
**Duration of symptoms, days**	1 (1–4)	1 (1–3)	.992
**Traumatic Etiology (%)**	32.2	41.4	.312
**Bilateral Pathology (%)**	13.5	17.2	.576
**Pathological Category (%)**			.955
Ocular Surface	54.2	55.2	
Eyelids and Orbita	22.7	24.1	
Control in systemic conditions^a^	11.6	7.0	
Visual Disturbances	2.7	3.4	
Other^b^	8.8	10.3	
**Most Accused symptoms (%)**			
Redness	36.9	27.6	.425
Discomfort	25.8	34.5	.280
Swelling	24.1	20.7	.823
Pain	16.1	17.2	.797
**Urgency (%)**	30.7	51.7	**.024**

^a^ Headache, fever, vomit or dizziness

^b^ Vitreoretinal diseases, neuro-ophthalmological conditions, glaucoma, uveitis, strabismus

## Discussion

This study shows a dramatic reduction of the PEED examinations carried out during the national lockdown in 2020. Compared to the average volume of visits performed in the same period in the previous three years, we experienced a drop of 79.4%. This finding is consistent with those reported by Shak et al., since they experienced a halving of the PEED consultations at their eye clinic during the first wave of the pandemic [6]. Conversely, our PEED has not direct access, so people have to stay in a shared waiting room before triaging and being sent to the Ophthalmic Unit. We hypothesized that the children’s parents, already discouraged by the restrictions related to the general lockdown, were even less inclined to attend a general pediatric ED, considering it a high-risk place for contracting the infection.

Another reason that has contributed to the reduction of PEED consultations was the interruption of those activities where children usually got injured, especially at school and during sport activities. Previous studies showed that PEED examinations due to ocular trauma represent from 13 to 38%, reaching a peak in spring and summer [7–10]. In accordance to the overall decrease, we have seen an absolute reduction in the number of PEED visits secondary to trauma (2017: 47, 2018: 41, 2019: 48, 2020: 12). Nevertheless, they represented a large part of the performed examinations and contributed to the significant rise of urgent cases (+ 21%) that characterized the CG. Compared to the pCG, the PEED was mainly attended by those children whose conditions required prompt assistance, reducing consistently the number of patients diagnosed with milder pathologies. A large amount of non-urgent cases could have been managed by pediatricians or ophthalmologists in telemedicine, or independently by parents.

No significant difference has been found in the sub-type of diagnosed pathology neither in the accused symptoms between the two periods and with respect to the common causes of request for consultation in PEED [11]. Anyway, during the lockdown, only one newborn was diagnosed with infective conjunctivitis, one of the most frequent conditions found in PEED before the pandemic. Schools closure and social distancing have contributed to the reduction of its incidence among adolescents and children, as well as other typical viral pathologies of the lower respiratory tract with air transmission [12].

It seems that parents, even during the emergency, didn’t use to wait before taking their children to a medical examination. This finding differs from what has been described regarding the adults’ behavior. Several studies, especially in non-ophthalmic settings, have reported that people perceived the hospitals as hotbeds for exposure and contamination and preferred to bear their symptoms rather than attending a hospital environment risking to get infected [13]. Even some patients affected by ophthalmic chronic pathologies have had a worse prognosis due to this reluctance or to the rescheduling of their programmed visits [4]. At the beginning of the COVID-19 emergency, when the disease wasn’t known enough, acute symptoms, even if urgent, had been overshadowed because the fear of contracting COVID-19 infection was higher than the experienced symptoms, but this did not occur in our pediatric sample.

## Conclusion

A significant drop in PEED consultations has occurred during the first wave of the COVID-19 pandemic. Although school and sports club closure was followed by an absolute decrease of eye examinations secondary to trauma, a great number of children with milder conditions has been self-managed by parents or pediatricians for their ophthalmic complaint. At the end of the emergency, better use of PEED could avoid overcrowding and minimize waste, allowing resource optimization for the management of urgent cases. Further studies are needed to assess the impact of the second wave of COVID-19 pandemic on the activity of PEED compared to the first one.

## Data Availability

Data are available from the corresponding author upon request.

## References

[1] Istituto Superiore di Sanità e Istituto Nazionale di Statistica. Impatto dell'Epidemia Covid-19 sulla mortalità totale della popolazione residente periodo Gennaio-Novembre 2020. Available at: https://www.iss.it/documents/20126/0/Rapp_Istat_iss_FINALE+2020_rev.pdf/b4c40cbb-9506-c3f6-5b69-0ccb5f015172?t=1609328171264. Accessed: December 30, 2020.

[2] Kabeerdoss J, Pilania RK, Karkhele R, Kumar TS, Danda D, Singh S (2021). Severe COVID-19, multisystem inflammatory syndrome in children, and Kawasaki disease: immunological mechanisms, clinical manifestations and management. Rheumatol Int.

[3] Toro MD, Brézin AP, Burdon M, Cummings AB, Evren Kemer O, Malyugin BE, Prieto I, Teus MA, Tognetto D, Törnblom R, Posarelli C, Chorągiewicz T, Rejdak R (2021). Early impact of COVID-19 outbreak on eye care: insights from EUROCOVCAT group. Eur J Ophthalmol.

[4] Song W, Singh RP, Rachitskaya AV. The effect of delay in care among patients requiring Intravitreal injections. Ophthalmol Retina. 2021. 10.1016/j.oret.2020.12.020. 10.1016/j.oret.2020.12.020 33395587

[5] Channa R, Zafar SN, Canner JK, Haring RS, Schneider EB, Friedman DS (2016). Epidemiology of eye-related emergency department visits. JAMA Ophthalmol.

[6] Shah K, Camhi SS, Sridhar J, Cavuoto KM (2020). Impact of the coronavirus pandemic on pediatric eye-related emergency department services. J AAPOS.

[7] Nelson LB, Wilson TW, Jeffers JB (1989). Eye injuries in childhood: demography, etiology, and prevention. Pediatrics..

[8] Sánchez Tocino H, Galindo Ferreiro A, Iglesias Cortiñas D, Galindo Alonso J, Fernández Muñoz M (2004). Epidemiologic study of ocular emergencies in a general hospital. Arch Soc Esp Oftalmol.

[9] Macewen CJ (1989). Eye injuries: a prospective survey of 5671 cases. Br J Ophthalmol.

[10] Voon LW, See J, Wong TY (2001). The epidemiology of ocular trauma in Singapore: perspective from the emergency service of a large tertiary hospital. Eye (Lond).

[11] Henríquez-Recine NS, Zafra B (2020). Ocular Emergencies in Children: Demographics, Origin, Symptoms, and Most Frequent Diagnoses. J Ophthalmol.

[12] Van Brusselen D, De Troeyer K, Ter Haar E (2021). Bronchiolitis in COVID-19 times: a nearly absent disease?. Eur J Pediatr.

[13] Boserup B, McKenney M, Elkbuli A. The impact of the COVID-19 pandemic on emergency department visits and patient safety in the United States. Am J Emerg Med. 2020;38(9):17321736–6. 10.1016/j.ajem.2020.06.007. 10.1016/j.ajem.2020.06.007 PMC7274994 32738468

